# Hybrid deep spatial and statistical feature fusion for accurate MRI brain tumor classification

**DOI:** 10.3389/fncom.2024.1423051

**Published:** 2024-06-24

**Authors:** Saeed Iqbal, Adnan N. Qureshi, Musaed Alhussein, Khursheed Aurangzeb, Imran Arshad Choudhry, Muhammad Shahid Anwar

**Affiliations:** ^1^Department of Computer Science, Faculty of Information Technology and Computer Science, University of Central Punjab, Lahore, Pakistan; ^2^Faculty of Arts, Society, and Professional Studies, Newman University, Birmingham, United Kingdom; ^3^Department of Computer Engineering, College of Computer and Information Sciences, King Saud University, Riyadh, Saudi Arabia; ^4^Department of AI and Software, Gachon University, Seongnam-si, Republic of Korea

**Keywords:** feature fusion, convolutional neural network, medical imaging, radiomics feature, deep feature

## Abstract

The classification of medical images is crucial in the biomedical field, and despite attempts to address the issue, significant challenges persist. To effectively categorize medical images, collecting and integrating statistical information that accurately describes the image is essential. This study proposes a unique method for feature extraction that combines deep spatial characteristics with handmade statistical features. The approach involves extracting statistical radiomics features using advanced techniques, followed by a novel handcrafted feature fusion method inspired by the ResNet deep learning model. A new feature fusion framework (FusionNet) is then used to reduce image dimensionality and simplify computation. The proposed approach is tested on MRI images of brain tumors from the BraTS dataset, and the results show that it outperforms existing methods regarding classification accuracy. The study presents three models, including a handcrafted-based model and two CNN models, which completed the binary classification task. The recommended hybrid approach achieved a high F1 score of 96.12 ± 0.41, precision of 97.77 ± 0.32, and accuracy of 97.53 ± 0.24, indicating that it has the potential to serve as a valuable tool for pathologists.

## 1 Introduction

In medical image analysis, object identification, detection, and recognition are essential skills that are applied in several settings, such as research, treatment planning, and illness diagnosis. Significant duties in this discipline include image registration, medical condition categorization, and tumor segmentation. In medical imaging, for instance, object detection entails locating and classifying anomalies, such as tumors, within an image. This is especially difficult because different medical problems present differently based on several circumstances, such as the patient's demographics and the imaging modalities used (Alruwaili et al., 2024; Wang et al., 2024). Conventional techniques for medical image analysis frequently depended on manually created features or textural attributes. The textural elements that Haralick et al. (1973) provided for image classification have been essential in understanding the textures observed in medical images. The Scale-Invariant Feature Transform (SIFT) is developed and Lowe (1999) finds unique local features in an image that are resistant to rotation, illumination, and scale changes. By producing a histogram of gradient orientations surrounding each key point, SIFT generates a descriptor which is then used to match key points across other images. Image registration and tumor localization in medical imaging have been made easier by the use of these descriptors for the recognition and alignment of anatomical components.

Convolutional neural network (CNN)-based object identification algorithms have significantly outperformed conventional object recognition algorithms in recent years as a result of the tremendous advancement in deep learning applications. These significant improvements have been noted by the medical image analysis field (Brahimi et al., 2017; Iqbal et al., 2024). Machine that understands features from raw images have gradually replaced algorithms that employ handcrafted features. Before AlexNet's innovation, numerous alternative methods for learning features were widely used (Bengio et al., 2013).

Three basic problems are specifically encountered while analyzing medical data, and these problems are briefly discussed below. Medical data may take on a variety of forms, from images to text values, and each type requires a particular approach. This presents many challenges when it comes to combining various types of data to, for instance, make a medical diagnosis (Yue et al., 2020; Xu Z. et al., 2023). The second issue is that conventional machine learning algorithms have performed poorly when used to analyze large amounts of data, particularly when it comes to the evaluation of medical data, which includes text notes and diagnostic images which is one of the primary medical tools used to estimate and inhibit human infection. Last but not least, a lot of medical data, including genetic expressions and bio-signals, have significant levels of noise and fluctuation, which enable information retrieval particularly challenging (Brahimi et al., 2018; Wang et al., 2023). Medical images are the primary diagnostic and prognostic tool utilized by physicians, which illustrates why statistical models are required for the interpretation of such material. Deep Learning might be viewed as the new approach in medical image interpretation. Medical images are generally utilized most frequently in the medical area of neuroscience for both brain-related studies and screening practices referred to neurological illnesses (Li et al., 2019). Such images are often distinguished by a significant level of diversity that may be controlled by complicated deep learning frameworks. These applications have demonstrated highly encouraging outcomes in the evaluation of such images in this context (Lin et al., 2020; Azam et al., 2022). Radiological examinations of the chest X-ray are often performed, and huge datasets have been used extensively in research to train algorithms that combine Recurrent Neural Networks (RNNs) for text interpretation and Convolutional Neural Networks (CNNs) for image analysis (Mavakala et al., 2023). Research on nodule recognition, description, and classification in radiography and thoracic computed tomography (CT) is still ongoing. Many strategies are being investigated (Xu Q. et al., 2023), including CNNs with conventional machine learning techniques and the integration of features produced from deep neural networks. These developments are intended to help organizations more effectively identify a variety of cancers on chest X-rays. Furthermore, the field of CT research endeavors to discern textural patterns suggestive of pulmonary ailments (Chen et al., 2023; Hao et al., 2023).

The clinical contemporary healthcare system has benefited greatly from the use of medical imaging. The ability of image segmentation technology to handle massive volumes of medical images has made it a crucial tool for computer-assisted medical evaluation and therapy. The medical image classification process is more difficult than conventional object recognition because of the subtle differences and higher levels of complexity in medical images. Additionally, the medical image contains a wealth of semantic data that is essential to clinical and pathological feature representation (Mohan and Subashini, 2018; Zhang et al., 2022). Low-level collection of features, mid-level feature visualization, and deep feature learning are the three main types of visual feature research used in medical image categorization. The low-level feature extraction methodologies, such as radiomics, SIFT (Lowe, 1999), Local Binary Patterns (LBP) (Liao et al., 2009), and Color Vector Patterns (Häfner et al., 2012) often characterize the image content primarily in terms of texture, form, color, and local pixel density. These approaches have a straightforward computation and a univocal notion but often typically omit semantic detail and are unable to deliver sufficient efficiency for a single feature. The low-level feature separation techniques are further used in the mid-level feature encoding approaches to provide statistics or acquisition that can do up to some extent and convey semantic significance. The widely used Bag-of-Visual-Words (BoVW) model, for instance, is still a useful feature representation method for environment image categorization (Nosaka et al., 2011).

Radiomics is a new branch of applied research that aims to extract high-dimensional data that may be mined from clinical imaging. The radiomic procedure may be broken down into discrete phases with defined sources and outcomes, such as image capture and reconstruction, image segmentation, feature collection and validation, assessment, and model creation. To build strong and trustworthy models that may be used in clinical applications for prediction, non-invasive disease monitoring, and assessment of disease reaction to therapy, each stage must be carefully assessed (Manthe et al., 2024).

A particular medical imaging technique cannot provide important precise and accurate results. This fact drives researchers to develop new imaging technologies or suggest fusion techniques to combine data from several manual feature extraction methodologies and acquire complementary information that may be present in one or more feature extraction methods (El-Gamal et al., 2016). Even if there are several medical image fusion methods, the clarity of the combined medical image may still be raised. To improve image resolution and expand the therapeutic application of images for medical concerns, the technique known as “image fusion” combines the more important information of numerous images from one or more feature extraction methodologies (James and Dasarathy, 2014; AlEisa et al., 2022). Image fusion techniques may be used at three distinct levels: feature, outcome-based, and pixel level. The goal of feature-level fusion techniques is to take the most important aspects or prominent features from the input images such as edges, direction, shape, and size. These prominent features are combined with other extracted features. A high level of fusion that determines the real target is called outcome-based fusion. It combines the output from many methods to get a final fusion assessment. With pixel-level fusion techniques, the actual data from the input images or their multi-resolution modifications is combined immediately (Liu et al., 2015).

According to a report by The International Agency for Research on Cancer (IARC) and The Global Cancer Observatory (GCO), it is measured that in 2020, there will be 308,102 new cases of brain tumor and 251,329 deaths worldwide (Sung et al., 2021). Among the various types of brain cancer, Glioblastoma Multiforme (GBM) is considered one of the most severe and challenging forms, which is classified as a grade IV brain tumor by the World Health Organization. It accounts for 48% of all initial malignant brain tumors and is expected to affect more than 13,000 Americans annually, with over 10,000 deaths each year, as reported by Bray in 2018. Treatment options for GBM are limited (Bray et al., 2018).

The goal of our study is to ascertain whether essential radiomics features might be present in various body cells. We evaluated the radiomics features of tumors in the brain organ. We created a Fusion model containing radiomics features and CNN features (high-level semantic features) extracted from available datasets for survival evaluation. The separate experimental dataset of brain cancers was used to examine the possibility of leveraging the chosen features to separate high-risk from low-risk groups. The study proposes using CNN and radiomics-based features to enhance the effectiveness of combined findings in medical image feature fusion. The main contributions of this study are as follows:

- A new residual block called Sequential-ResNet (Seq-ResNet) is proposed, which includes five 3 × 3 convolutional layers to examine high-level semantic information. The proposed Seq-ResNet deepens the CNN network while maintaining a manageable parameter number and adds an approach to preserve moderate-level features in addition to shortcut connections.
- A FusionNet architecture with 37 layers is designed specifically for detecting smaller or low-magnification tumors, which allows for the combination of high-level semantic information with moderate-level pertinent features.
- Radiomics features can be obtained from the tumor region that has been segmented using image processing techniques. Various feature extraction methods, including gray-level co-occurrence matrix (GLCM), gray-level run length matrix (GLRLM), and gray-level size zone matrix (GLSZM), are available for this purpose.
- Feature fusion is used to extract the most discriminating information from the source feature sets and eliminate duplicate information produced by association across different feature sets.
- The statistical and spatiotemporal features in each of the various source images are extracted using an inter-extraction method, combined, and then divided into malignant and benign.

This research employs the medical imaging modality MRI, to detect brain tumors. Brain MRI is widely used for diagnosing critical diseases globally, and Section 2 describes the details and prevalence of such diseases. Section 3 reviews the most up-to-date state-of-the-art research on brain MRI. Section 4 discusses the importance and significance of this article, while Section 5 elaborates on our proposed fusion model's detailed design and implementation. The experimental setup is presented in Section 5.3. Finally, Section 6 details the results obtained from training and testing on the Brain MRI dataset, and Section 8 concludes the study.

## 2 Background

According to the American Cancer Society, negative consequences of GBM therapy may include peripheral neuropathy, which includes symptoms that cause effects on the central nervous structure and limit bodily activities, lowering the quality of life for patients severely. As a result, it is vital to determine if chemotherapy will be beneficial in slowing the progression of the disease before the patient begins treatment.

Surgical excision of the tumor, followed by radiation and chemotherapy, is the treatment option for GBM patients. Individual patients who get standard care have an average survival period of 15 months, relative to only 4 months if they are left untreated once identified (Bleeker et al., 2012). Chemotherapy, a common and effective therapeutic option, kills rapidly proliferating cells but cannot always ensure the difference between tumor and normal cells. This may have unfavorable consequences (Taal et al., 2015).

One of the most challenging jobs in medical imaging analysis is the automatic classification of glioma. It would be very helpful for healthcare professionals if a computational framework could be created that could detect diseases, plan treatments, and evaluate their effectiveness better than a trained and qualified one could. Such a framework would also enable a more distinct, uniform, and exchangeable method for diagnosis of diseases, care planning, and measurement. Gliomas are the most prevalent type of brain tumor in people. The appropriate classification of medical image data is a provocative medical image analysis job because of their complex structure and composition in multi-modal Magnetic Resonance Imaging (MRI). Such gliomas require feature extraction, which requires a high level of specialist knowledge, requires time, and is inclined to human misconception. The typical approach also deficiency of coherence and reproducibility, which has a negative effect on the results and may result in incorrect diagnosis and treatment.

Due to rapid advancements in machine learning and deep learning (DL) techniques, deep neural networks (DNNs) hold a lot of potential for application in Computer Assisted Diagnosis (CAD) semi-automatic systems for healthcare data interpretation. Convolutional neural networks (CNNs) have significantly advanced, enabling models to match or surpass human performance in a variety of fields, including, among others, image analysis and microscopy segmentation (Russakovsky et al., 2015).

Despite the apparent effectiveness of Deep learning models in a variety of problem scenarios, designing well-functioning deep learning models is not easy in reality. The success of a deep learning model is strongly dependent on a circumstantially appropriate selection of design factors, such as the number of hidden layers in a model, the number of units in a layer, and the kind of unit, which are referred to as hyperparameters. Different elements of the deep learning model's behavior are governed by hyper-parameters, including the model's capacity for learning patterns from images, its degree of generalization in performance when given with fresh data, and the memory consumption cost of building the classifier (Nazir et al., 2022).

As we all know, deep learning models are black boxes and we do not know about the pertinent feature extractions and also these models are data hungry. As long as enough training data are provided, deep learning models are strong contenders for brain malignancy segmentation. The Brain Tumor Segmentation Challenge (BraTS) offers a huge, eminent-quality dataset that includes MRI brain images and segmentation masks. Tumor segmentation and MGMT methylation prognosis from pretreatment magnetic resonance (MR) images are two challenges in the RSNA BraTS 2021 competition. The organizers of the challenge have published large datasets to facilitate technique assessment and advance state-of-the-art approaches in various fields (Baid et al., 2021).

Due to the tiny size of medical image segmentation datasets (typically ~100 samples) and the lack of a universal baseline for comparing the effects of different architectural adjustments, such analyses are frequently incorrect. Nevertheless, the dataset published for BraTS21 contains 2,040 samples (in the training, validation, and test sets, respectively, 1251, 219, and 570 examples), making it the leading dataset for medical image analysis at the instant and an ideal individual for comparing performance improvements for different UNet variants.

There has subsequently been a surge in the field of information mining and artificial intelligence (AI) usage in medicine. The topic of radiomics encompasses a set of approaches for automatically extracting huge quantities of statistical data from medical images using gray-level pixel assessment, potentially paving the way for discoveries into pathophysiological processes underpinning various medical disorders (Lambin et al., 2012). One of the key fields of radiomics is texture characterization, which evaluates gray-level value variations in images that are not discernible by a human reader's aesthetic judgment. As a result, it is useful in radiography for assessing the characteristics of various tissues or organs, perhaps leading to the discovery of novel biomarkers (Scalco and Rizzo, 2017). Texture features and characteristics may have clinical and pathological associations that might aid in the assessment of patient prognosis (Lubner et al., 2017).

Deep Learning (DL) has proven to be potentially effective in a variety of healthcare sub-specialties in recent years, and many of these techniques have now been licensed for clinical usage (Cuocolo et al., 2019, 2020; Tsuneta et al., 2021). Radiology is one of the most promising domains for radiomics and machine learning applications, since they may be used to detect and characterize lesions automatically or divide medical images (Ugga et al., 2021; Spadarella et al., 2022). There have been an increasing number of research studies that indicate DL to be a valuable technique in visualizing malignant disorders (Haq et al., 2021). It might, for example, reduce the time it takes to acquire and rebuild images (Sermesant et al., 2021). The have also shown encouraging results in digital anatomical structure segmentation and illness categorization (Bruse et al., 2017; Ghorbani et al., 2020). Finally, the capacity of DL to find hidden patterns in data may bring fresh insights into well-known illnesses, boosting future management (Bagheri et al., 2021).

## 3 Related work

Quan et al. (2021) presents FusionNet, a deep neural network that segments neuronal structures in connectomics data obtained from high-throughput, nano-scale electron microscopy. The primary challenge of developing scalable algorithms with minimal user input is addressed with deep learning. FusionNet combines recent machine learning advancements to improve segmentation accuracy and performs well when compared with other electron microscopy segmentation techniques. The versatility of FusionNet is also demonstrated in two segmentation tasks: cell membrane segmentation and cell nucleus segmentation.

Guo et al. (2020) proposed framework for multi-modal medical image integration, which aims to maximize physiological information, improve visual clarity, and reduce computation. It consists of four parts and captures all medical information features in the input image, calculates the weight of each feature graph, and reduces information loss. The algorithm was tested on three sets of investigations with medical images, showing better performance than other algorithms in terms of detail and structure recognition, visual features, and time complexity.

The deepSeg was discovered by Zeineldin et al. (2020). They created two fundamental components that are linked by an encryption and decoding connection. To extract features, they employed a Convolutional Neural Network (CNN) as an encoder. With CNN layers, they employed dropout and Batch Normalization (BN). Then, using the SoftMax activation function, enter the result into the decoding section to generate a prediction map. They employed a Batch Normalization layer between each convolution and ReLU in the deciphering section and a modest kernel size of 32 for the base filter. They also examined the revised UNet to other CNN models including NASNet, DenseNet, and ResNet. They used FLAIR MRI images from the BraTS 2019 competition, which enclosed 336 training instances and 125 validation cases for data size of 244 × 244. Hausdroff and Dice's Distances increased from 0.81 to 0.84 and 9.8 to 19.7, respectively.

For brain tumor segmentation, Lachinov et al. (2018) identified two frames of classification techniques from the same UNet (Multiple Encoders UNet and Cascaded Multiple Encoders UNet). For brain segmentation, they employed a customized 3D UNet CNN Model. With its cost function, the proposed Cascaded UNet utilized three UNets. To improve the dataset, they employed z-score normalization as a pre-processing strategy. To expand the range of instances of the source data, they employed b-spline transformation as data augmentation. They tested the proposed two-frame classification techniques using the BraTS 2018 dataset and found that they performed well on test data. The Dice score increased from 0.901/0.779/0.837 to 0.908/0.784/0.884 for total tumor, enhanced tumor, and tumor core segmentation when the base UNet was evaluated to the Cascaded UNet.

Based on the number of references, encoder–decoder architectures, in particular UNet, are among the most often used deep learning models for medical image analysis in the field of brain tumor segmentation (Ronneberger et al., 2015). UNet-like topologies have been among the most popular BraTS competition proposals in recent years. For example, in 2018, Myronenko added a variational autoencoder branch to a UNet model for generalization (Myronenko, 2018). Jiang et al. (2019) used a two-stage UNet pipeline to partition brain tumor structural components from rough to granular in 2019. Isensee et al. (2021) reported the nnUNet architecture in 2020, with particular BraTS-designed improvements to data post-processing, region-based training, data augmentation, and minor nnUNet flow improvements (Isensee et al., 2020). These results demonstrate that well-designed UNet-based networks may execute well enough on challenges such as brain tumor segmentation. To develop a suitable solution for problems such as BraTS21, the optimum neural network design, and the training procedure must be adopted. There are many other types of UNets, such as Attention UNet (Oktay et al., 2018), Residual UNet (He et al., 2016), Dense UNet (Huang et al., 2016), Inception UNet (Szegedy et al., 2017), UNet++ (Zhou et al., 2018), SegResNetVAE (Isensee et al., 2020), or UNETR (Hatamizadeh et al., 2022), to mention a few.

We examine feature matching encoder–decoder systems from two angles: reconstructing spatial features and utilizing hierarchical semantics. The pooling mechanism in encoder–decoder networks is notorious for inducing significant systematic errors and overlooking the connection between parts and wholes. In convolutional neural networks (CNNs), max-pooling is frequently used for downsampling. The greatest value from each region is generated by max-pooling, which divides feature maps into non-overlapping parts. This results in the loss of potentially significant geographical information. Several existing strategies have attempted to modify crude high-level semantics through the use of high-level spatial resolution information. In combination with multiresolution fusion, stacked hourglass networks perform continuous bottom-up and top-down computation (Newell et al., 2016). Recent approaches append the characteristics of various layers before prediction calculation to retrieve spatial information employing encoder–decoder networks (Bell et al., 2016; Kong et al., 2016). As the input to other concurrent sub-networks, HRNet integrates the representations created by sub-networks with high-level resolution (Sun et al., 2019). Deeply fused networks employ shallow layer interim outputs as input to deeper layers (Chen et al., 2021). The global convolutional network uses skip connections with massive kernels to encode rich spatial information from input images (Peng et al., 2017).

The high-level interpretations heavily influence the outcome of an encoder–decoder network. However, feature merging is necessary to restore low-level semantics in addition to high-level spatial characteristics. To prevent unnecessary failed states that can result from increasing depth, ResNet adds low-level semantic input feature maps to high-level semantic output feature maps (He et al., 2016). In contrast, DenseNet combines hierarchical semantics with spatial information at the same level, thereby improving classification rules (Huang et al., 2017). H-DenseUNet showcases how the optimized flow of information and parameters can reduce the complexity of training encoder–decoder networks for biomedical image segmentation (Li et al., 2018).

Badrinarayanan et al. (2017) discovered a convolutional encoder–decoder network for image analysis in their study known as SegNet. The SegNet is a fundamental trainable segmentation engine that includes an encoder network, which is structurally similar to the VGG-16 network's 13 convolutional layers, and a corresponding decoder network, along with a pixel-wise classification layer akin to the deconvolution network. What sets SegNet apart is its innovative approach to non-linear upsampling in the decoder, where it employs the pooling indices obtained during the associated encoder's max-pooling phase. This eliminates the need for learning how to up-sample. To produce dense feature maps, trainable filters are used to convolve the up-sampled (sparse) maps. SegNet outperforms many of its competitors while requiring a considerably smaller number of learnable parameters. The same authors proposed a Bayesian version of SegNet to model the uncertainty in the convolutional encoder–decoder network for scene segmentation (Kendall et al., 2015).

In their groundbreaking study, Aerts et al. (2014) reported predictive ability in separate data sets of individuals with head-and-neck and lung cancer. It showed that frequently obtained CT scans may contain diagnostic and biological information. As a result, a significant amount of radiomics research has concentrated on this topic after acknowledging that tumor diversity has prognostic value and may affect therapy response (McGranahan and Swanton, 2015). The relevance of radiomics for diagnosis and prognosis and evaluating therapeutic outcomes is highlighted by research that proves radiomics characteristics and patterns mirror the cancer micro-environment in terms of behavior and progression. For patients with non-small cell lung cancer, Ganeshan et al. (2012) discovered that tumor variability may be evaluated by non-contrast CT scan texture analysis and can offer an unbiased predictor of survival (NSCLC). The same researchers' texture characteristics found pertinent relationships in a different investigation and showed that they might function as imaging correlates for cancer hypoxia and angiogenesis (Ganeshan et al., 2013). Win et al. (2013) used pretreatment Positron Emission Tomography (PET)/CT scans to study cancer heterogeneity and permeability throughout this time. According to their research, the only variable associated with survival in the group receiving drastic therapy was textural heterogeneity assessed from CT scans. Textural heterogeneity, tumor stage, and permeability were all linked to survival outcomes in the palliative treatment group. In a similar setting, Fried et al. (2014) retrieved texture characteristics from preoperative CT images before receiving final chemo-radiation therapy and discovered that radiomics features may offer predictive information further than that gained from standard prognostic markers in NSCLC patients. Based on the widely accepted idea that tumors are diverse and the degree of diversity may aid in determining the malignancy and severity of tumors, Cherezov et al. (2019) discovered a method for discovering tumor habitats using textural data. These findings showed that lung cancer patients' long-term and short-term survival rates could be distinguished with an AUC of 0.9 and an accuracy of 85% (Cherezov et al., 2019). Furthermore, previous research has shown correlations between prognosis and therapeutic response for radiomics characteristics derived from preoperative fluorodeoxyglucose (18F-FDG) PET scans. For instance, in a previous study, textural characteristics of PET scans were linked to worse prognostications and lack of response to chemo-radiotherapy by Response Evaluation Criteria in Solid Tumors (Cook et al., 2013). Decreased diversity on PET was linked to erlotinib reaction in a different research by Cook et al. (2015), and changes in first-order entropy were significantly linked to both patient survival and effective manner and response in NSCLC patients. They explain the clustering strategy using FDG-PET and CT to detect intra-tumor diversity in lung adenocarcinomas before and after therapy. To quantify the lesion structure, strength, diversity, and other characteristics in several frequencies, 583 radiomics characteristics from 127 preoperative lung nodules were retrieved in a different research. With equal-sized benign or malignant tumors, patients were randomly assigned to one of ten categories. The random forest approach was then used to run a diagnostic model. This radiomics classification algorithm successfully achieved 80.0% sensitivity, 85.5% specificity, and 82.7% accuracy in separating cancerous primary lesions from benign ones. In contrast, the sensitivity of the conventional knowledgeable radiologists' annotations was only 56.9% with identical precision (Ma et al., 2016). Another study reported how radiomics may identify the eventual development of cancer by doing quantitative analysis on preliminary low-dose CT chest lesions and analyzing images from the well-known National Lung Screening Trial (NLST). There were two lineages: one included 104 cases and 92 individuals with lung malignancies found by screening, while the other included a similar group of 208 incidents and 196 individuals with harmless pulmonary lesions found through screening. Such findings are comparable to the precision of the McWilliams framework for analysis and outperformed the accuracies of the Lung-RADS and malignancy volume methodologies (Kim et al., 2019). In total, 23 reliable radiomics features chosen by the Random Forest (RF) algorithm accurately predicted malignancy that became malignant in 1 or 2 years, with accuracies of 80% (Area Under the Curve, AUC 0.83) and 79% (AUC 0.75), respectively (Hawkins et al., 2016). Using peritumoral and intra-tumoral radiomic characteristics, Pérez-Morales et al. (2020) created an independent lung cancer prognostic prediction model (Pérez-Morales et al., 2020). This algorithm might pinpoint a subset of individuals with initial lung cancer who are at significant risk and have a bad outcome. The lung cancer screening system allowed doctors to customize clinical care for these high-risk individuals who were diagnosed with lung cancer in its early stages. Furthermore, Horeweg et al. (2014) identified that determining the radiomic capacity doubling time for medium lesions helped direct lung cancer care and perhaps forecast the likelihood of lung cancer (Horeweg et al., 2014). Lesion treatment strategies that use volumetric or volume-based diameter boundaries (ranging from 9 to 295 mm^3^ or 6–11 mm in diameter) have demonstrated enhanced sensitivity of up to 92.4% and specificity of 90.0% as opposed to the ACCP lesion handling procedure using low-dose CT scans in target populations. By lowering false positives and negatives in lung cancer analysis and misdiagnoses, more research into radiomics applications could improve lung cancer screening. Constanzo et al. (2017) has generally concentrated on hand-crafted Radiomics, while deep learning-based Radiomics is only briefly discussed without addressing various topologies, interpret-ability, and hybrid models. While Parmar et al. (2018) discovered both forms of Radiomics, the combination of hand-crafted and deep learning-based characteristics is not taken into account. Additionally, the difficulties with radiomics and the connection between radiomics and gene expression (radio-genomics) are not fully covered. Finally, only deep learning-based radiomics features are covered in the study by Litjens et al. (2017), leaving out hand-crafted features, their stability, hybrid radiomics, and radio-genomics. All of them necessitate a quick and timely effort to expose radiology to your community, as image processing is one of the fundamental elements of radiology.

The entropy of metastatic disease was proven to be a relevant measure in an MRI-based radiomics investigation; greater entropy values were discovered in tumor tissues relative to mild tumors, indicating the tumor's diversity and vascular state (Parekh and Jacobs, 2017). Using dynamic contrast-enhanced MRI, another research (Whitney et al., 2019) attempted to develop a collection of quantitative parameters that might be retrieved from MR images to differentiate luminal breast tumors from mild breast tumors.

## 4 Our contributions

When Deep Convolutional Neural Networks (DCNNs) utilize feature fusion for retrieving spatial information and leveraging multi-layer semantics, two issues arise. First, the deep convolution layer feature maps provide lower-level spatial information required for reconstructing the merged feature maps. Second, feature-matching methods only provide feature maps' semantics at the same level of resolution. These problems are challenging to address as element-wise addition and channel concatenation result in a fusion method that is overly restrictive and only aggregates extracted features of the same scale. The encoder and decoder convolution layers are the only ones at the same level in encoder–decoder networks because downsampling reduces the scale of feature maps, and upsampling increases it. UNet lacks multi-layer semantics and global spatial information, which results in analyzing images pixel by pixel and distinguishing objects using color contrast. However, using contrasting colors to make objects stand out may not necessarily improve tumor borders.

The purpose of our investigation is to determine whether fundamental radiomics traits may be found throughout several human tissues. In the brain organs, we assessed the radiomics characteristics of malignancies. Using the dataset for survival analysis, we built a radiomics model and selected characteristics. The potential for employing the desired traits to distinguish between high and low-risk groups was investigated using an independent test dataset BraTS brain tumors.

To achieve automation with manual medical image feature fusion, we use CNN and radiomics-based features to upgrade the effectiveness of the combined findings. The main contributions of this study are as follows:

1. Sequential-ResNet (Seq-ResNet) is a new residual block, which is proposed. Five 3 × 3 convolutional layers are included in a single Sequential-ResNet to examine high-level semantic information. A hierarchical framework made up of these five layers generates features that are then analyzed by different numbers of convolutional layers. The proposed Seq-ResNet deepens the CNN network while maintaining a manageable parameter number as compared with the original ResNet. The proposed Seq-ResNet adds an approach to preserve the moderate-level features in addition to the shortcut connection increasing the outcomes.
2. We design a Seq-ResNet for the detection of a smaller or low-magnification tumor. This CNN architecture comprises 37 Seq-ResNet layers. The proposed model having a deeper architecture allows the model to capture more intricate and complex high-level semantic features from the input data. Additionally, the pairing of numerous Seq-ResNet preserves the moderate-level features necessary for smaller tumor recognition while allowing the resulting features to be digested by varied numbers of convolution layers. As a result, the feature produced by the proposed model combines high-level semantic information with moderate-level pertinent features.
3. The essence of feature fusion lies in its ability to extract the most discriminating information from the source feature sets engaged in fusion and get rid of the duplicate information produced by association across different feature sets. The statistical and spatiotemporal features in each of the various source images are extracted using an inter-extraction method. The source images' features are combined, and the combined result is divided into malignant and benign.

## 5 Methodology

### 5.1 FusionNet

The natural contour of the body edge is changeable in medical images because distinct features of the human body are visible. To handle the separation of microorganisms, a structure has been developed that uses sophisticated models which amalgamate a high-level representation of depth-wise convolution layers and residual blocks with a description of the presence of upsampling and downsampling levels to achieve detailed segmentation. The original residual block is suggested as a solution to the vanishing gradient issue that arises as network complexity grows. Networks with different depths have been developed to effectively investigate high-level contextual information by layering numerous residual blocks. Low-level precise information is observed as being as significant as high-level contextual information in the case of tiny feature extraction. Although the auxiliary link in the initial residual block aids in deepening the network, convolutions still lead to low extensive information vanishing. The 3 × 3 convolution layer in the original residual block is replaced with a sequential ResNet convolution structure to allow for simultaneous acquisition of low-level specific information and high-level contextual information. Proposed Seq-ResNet is the term given to the res-block since the input feature is separated and analyzed hierarchically, which is similar to mounting a mountain.

**Figure 1:**
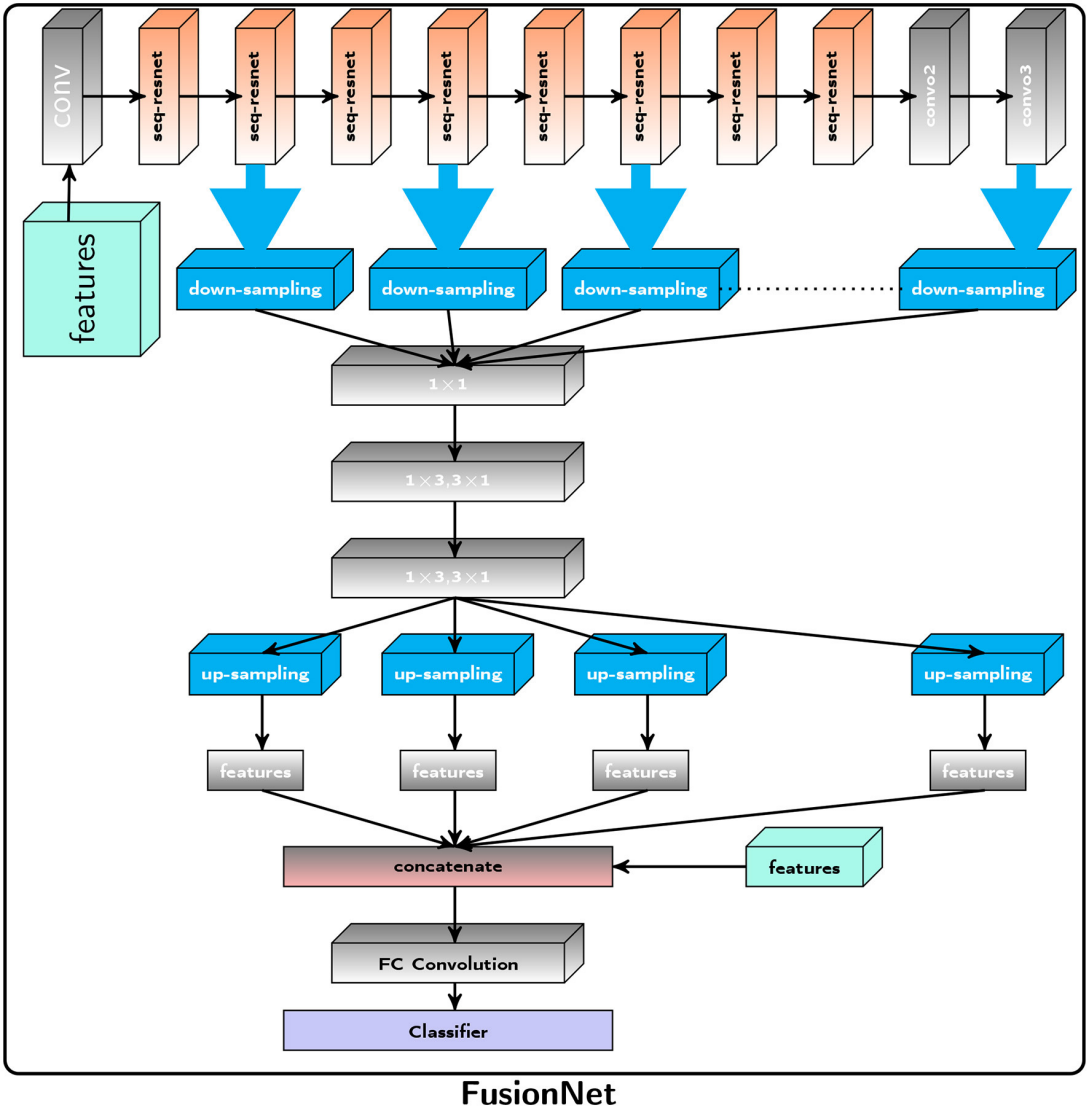
Proposed Framework: The Proposed FusionNet, where seq-resnet layer is shown in Figure 2, extracts feature subspace (3 × 3), represents the number of convolution layers, the subspace has gone through, and j represents the subspace number. The extracted features via Seq-ResNet are forwarded for downsampling steps and 1 × 1 convolution to 1 × 3, 3 × 1 depth-wise separable convolution, respectively. After that, we apply upsampling to get the pertinent features, and these features are concatenated with radiomics features and apply the FC layer. These concatenated features are passed to the Support Vector Machine (SVM) for classification.

**Figure 2:**
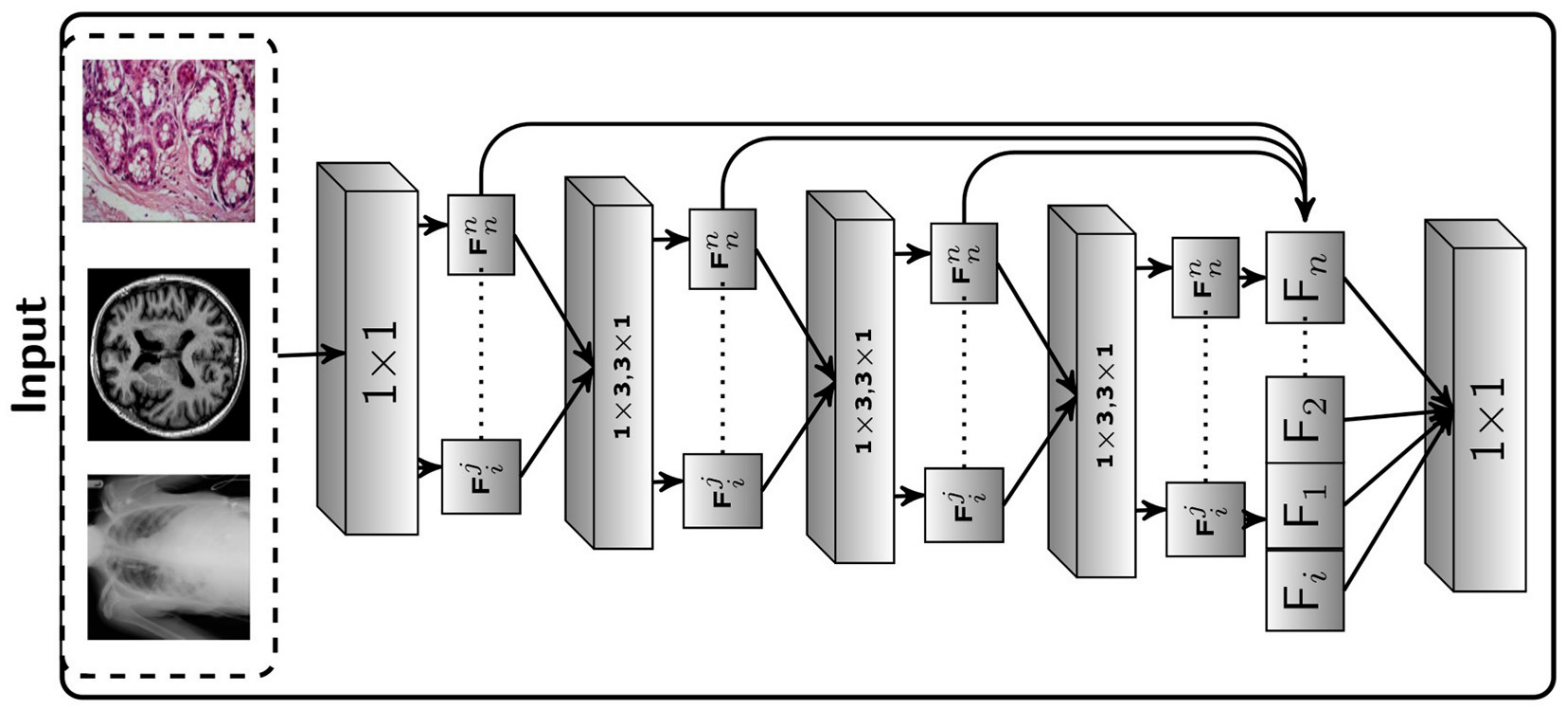
Proposed Framework: The suggested Seq-ResNet, where ${𝔽}_{i}^{j}$ represents a feature subspace, i(3 × 3) represents the number of convolution layers the subspace has gone through, and j represents the subspace number. 𝔽_*i*_ denotes a feature map built from feature subspace (i indicate the number of this feature map). The symbol ${\mathbb{C}}_{i}^{j}$ represents a convolution layer, with *j* representing the kernel value and *i* representing the number of layers. The Seq-ResNet input image is convolved with kernel 1 × 1 to reduce the number of channels to $\frac{1}{5}$. These numbers of channel output such as ${𝔽}_{1}^{1}$, ${𝔽}_{2}^{1}$, ${𝔽}_{3}^{1}$, and ${𝔽}_{4}^{1}$, are convolved with kernel value 3 × 1 and 1 × 3 except the ${𝔽}_{0}^{1}$.

The Proposed FusionNet algorithm is shown in Algorithm 1 and Figure 1 and use of Seq-ResNet is shown in Figure 2 to obtain feature subspace, where j is the subspace number and (3 × 3) is the size of convolution layers the subspace has undergone. The Seq-ResNet-extracted features are then sent forward for downsampling steps and 1 × 1 depth-wise separable convolution with 1 × 3 and 3 × 1, respectively. The relevant characteristics are then obtained by using upsampling, and these features are combined with radiomics features before applying an FC layer. Support Vector Machine (SVM) is given these concatenated characteristics to classify the data. The proposed CNN model FusionNet comprises Seq-ResNet and is made up of two parts:

**Algorithm 1:**
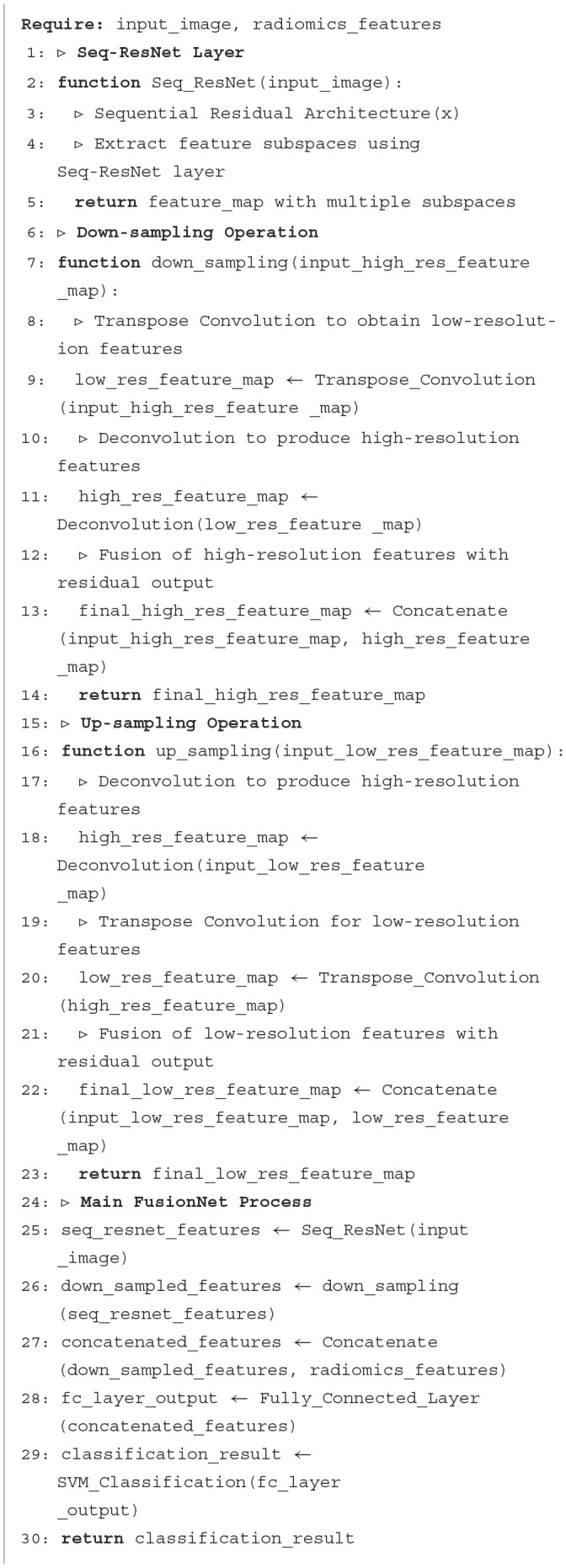
Proposed FusionNet.

#### 5.1.1 Sequential residual network

Figure 2 depicts the proposed Seq-ResNet, where ${𝔽}_{i}^{j}$ stands for a feature subspace (*i* [3 × 3] for the number of convolution layers the subspace has undergone and *j* for the subspace number). 𝔽_*i*_ stands for a feature map constructed from feature subspace (*i* indicates the number of this feature map). The symbol ${\mathbb{C}}_{i}^{j}$ denotes a convolution layer with *j* depicting the kernel value and *i* presenting the number of layers. The input image of Seq-ResNet is convolved with kernel 1 × 1 to decrease the number of channels into $\frac{1}{5}$. These number of channel outputs such as ${𝔽}_{1}^{1}$, ${𝔽}_{2}^{1}$, ${𝔽}_{3}^{1}$, and ${𝔽}_{4}^{1}$ are convolved with kernel value 3 × 1 and 1 × 3, except the ${𝔽}_{0}^{1}$. The remaining feature (${𝔽}_{0}^{1}$) is processed-free overlaid to final feature map (𝔽_*i*_). We then pass ${𝔽}_{2}^{1}$, ${𝔽}_{3}^{1}$, and ${𝔽}_{4}^{1}$ to depth-wise seperable convolution with kernel value 3 × 1 and 1 × 3, and the remaining feature ${𝔽}_{0}^{1}$ and ${𝔽}_{1}^{1}$ forward to final feature map (𝔽_*i*_). The detailed operation is shown in Figure 2 Seq-ResNet block. At the end, we concatenate all the processed and processed-free features into a final feature map 𝔽_*i*_. To restore the original channel number, we process the concatenated feature map with convolution with a kernel value is 1 × 1. Multiple subspaces of the result are analyzed using various convolutional layers, resulting in various subspaces that include features with various receptive fields. Smaller receptive field subspace, such as ${𝔽}_{0}^{1}$ and ${𝔽}_{1}^{1}$, comprise more relevant details and undergo smaller convolution stages, which is crucial for smaller tumor recognition. Large receptive field subsets, such as ${𝔽}_{2}^{2}$, ${𝔽}_{3}^{3}$, and ${𝔽}_{4}^{4}$, blur particular information while exploring deep contextual features is equally crucial for detection. At the start and conclusion of the suggested block, two ${\mathbb{C}}_{1}^{3}$ and ${\mathbb{C}}_{2}^{3}$ are utilized to automatically choose the appropriate features and apply a bottleneck to lower the parameter count.

#### 5.1.2 Upsampling and downsampling

**Figure 3:**
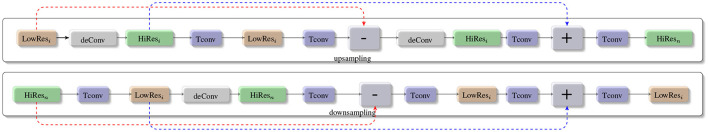
The low resolution feature maps (LowRes*i*) are passed to deConvolution to produce the high resolution features (HiRes*i*). The Transpose Convolution (Tconv) algorithm is used to extract relevant features from low resolution (LowRes*i*) images and convert them to high resolution (HiRes*i*). The residual outcome of the low resolution (LowRes2) feature map and the initial low resolution (LowRes1) feature map is passed to deConvolution to produce high resolution (HiRes*i*) feature maps. The final high resolution residual output is obtained by concatenating the output of the Transpose Convolution with the output of the initial high resolution feature map (HiRes1). In the downsampling technique, we send high resolution (HiRes*i*) feature maps to Transpose Convolution to obtain low resolution (LowRes*i*). These residual results are deconvolved to produce high resolution feature maps (HiRes*i*). The preliminary high resolution feature maps are fused with the residual output of the second Transpose Convolution, which is applied to the second high resolution feature maps. Following concatenation, we use Transpose Convolution to create low resolution feature maps (LowRes*i*).

Typically, a compilation of feature maps is created by sampling and concatenating the information from various levels of the proposed Seq-ResNet. To construct a feature selection procedure, several 1 × 1 convolutions are applied during the gathering of feature maps. After feature extraction, the gathered features are merged to create feature maps with various resolutions, which are then put through distinct upsampling and downsampling procedures. Throughout the upsampling and downsampling operations, both high and low contextual information from the proposed feature fusion network can be used. However, because of these processes, information impurity might happen. Our suggested feature fusion network uses a deep upsampling and downsampling deConvolution layer to address this problem. This lessens the effect of imperfect information. This methodology was motivated by the deep upsampling and downsampling units found in super-resolution image reconstruction methods. Figure 3 depicts the proposed deep upsampling and downsampling deConvolution module's structure. The two main components, upsampling and downsampling, are used to extract low-level and high-level contextual features. The upper layer of Figure 3 presents the upsampling methodology. The low-resolution feature maps (LowRes_*i*_) are passed to deConvolution to produce the high-resolution features (HiRes_*i*_), as shown in Figure 3. The Transpose Convolution (Tconv) is used to extract pertinent features from low resolution (LowRes_*i*_) to convert it into high resolution (HiRes_*i*_). The low resolution (LowRes_2_) feature map residual outcome and initial low resolution (LowRes_1_) feature map are passed to deConvolution to obtain high resolution (HiRes_*i*_) feature maps. The final high resolution residual output is obtained from the initial high resolution feature map (HiRes_1_), and the output of the Transpose Convolution is concatenated in final high-resolution feature maps (HiRes_*n*_). The lower layer of Figure 3 portrays the downsampling technique. Initially, we pass high resolution (HiRes_*i*_) feature maps to Transpose Convolution to acquire low resolution (LowRes_*i*_). These residual outcomes are passed to deConvolution to get the feature maps of high resolution (HiRes_*i*_). The initial high resolution and the residual output of the second Transpose Convolution which are applied on second high-resolution feature maps are fused. After concatenation, we apply Transpose Convolution to generate the low-resolution feature maps (LowRes_*i*_). These low resolution feature maps are convolved with Transpose Convolution and then fused with the initial low resolution feature maps. After fusion, we apply Transpose Convolution to achieve low-resolution residual feature maps.

The term “handcrafted features” in our study refers to certain traits or attributes that are retrieved from raw data by following predetermined rules or algorithms. Other names for these characteristics include “handmade features” and "hand-engineered features." They are thoughtfully created and defined by researchers or domain specialists based on their comprehension of the data and the particular issue being addressed. In our study, we use a set of statistical and textural metrics that we extract from medical tomography images as these “handcrafted features.” The choice of these metrics is an important step since they help obtain vital information about the texture and characteristics contained in the images.

These hand-crafted features include metrics derived from various statistical approaches and texture analysis methods, gradient-based features, and other characteristics. The Gray Level Co-occurrence Matrix (GLCM), the Gray Level Run Length Matrix (GLRLM), and the Gray Level Size Zone Matrix (GLSZM) are three well-known matrices that we specifically add to our features. With the aid of these matrices, we can record vital details on the connections between pixel values and their spatial distribution within the images.

#### 5.1.3 Radiomics features

The qualities of radiomics features, such as histograms, textures, shapes, transformations, and models, may be categorized numerically (Benoit-Cattin, 2006). Radiomics features may be retrieved from either 3-dimensional (3D) Volumes of Interest (VOIs) or 2-dimensional (2D) Region of Interest (ROIs). We used ROI as a catch-all word for both to make the text easier to read. Additionally, gray-level brightness that has not been changed or discretized can have numerical features estimated. Gray-level discretization and feature importance aggregation are not covered in this article since they fall outside of its purview. Gray-level discretization limits the range of gray levels to a predetermined number to improve reliability and manageability, whereas feature significance aggregation obtains a single value when the same feature is recognized in various forms and simplifies it using average values. Gray-level variance, minimum, maximum, mean, and percentiles are the most basic statistical variables that are derived on the global gray-level histogram (Zwanenburg et al., 2020). First-order features are those that are dependent on single-voxel or single-pixel analysis. Skewness and kurtosis are more complex characteristics as depicted in Equation (1) which describe the brightness distribution of the data. Skewness must represent the leftward or rightward asymmetry of the data distribution curve (negative skew, below the mean) (positive skew, above the mean). Kurtosis tends to reflect the tailedness of a distribution of the data compared with a Gaussian distribution as a result of anomalies. Other characteristics include energy also called homogeneity and histogram entropy. These are distinct from their corresponding co-occurrence matrix models of the same name.

By measuring the intensity variations in gray levels throughout an image, studying the absolute gradient provides a straightforward method for characterizing genuine radiomics textures. When two adjacent pixels or voxels have the same color, the gradient stays at zero, and it reaches its maximum when one is black and the other is white or vice versa. Similar to histograms, gradients are subjected to statistical features including variance, skewness, kurtosis, and mean, regardless of the direction of the gray-level transition (Benoit-Cattin, 2006; Zwanenburg et al., 2020). To balance the ratio of the dataset, we apply preprocessing techniques and data augmentation methodology before retrieving radiomics features.


(1) $$\begin{array}{l} \text{Sk}=\frac{n}{\left(n-1)(n-2\right)}\sum_{i=1}^{n}{\left(\frac{x_{i}-\bar{x}}{s}\right)}^{3}\\ \text{K}=\frac{n(n+1)}{\left(n-1)(n-2)(n-3\right)}\sum_{i=1}^{n}{\left(\frac{x_{i}-\bar{x}}{s}\right)}^{4}-\frac{3{\left(n-1\right)}^{2}}{\left(n-2)(n-3\right)} \end{array}$$

In their research, Haralick et al. (1973) established the second-order gray-level histogram feature known as the Gray Level Co-occurrence Matrix (GLCM), as shown in Equation (2). The spatial associations between pairs of pixels or voxels with defined distances between them, predetermined gray-level intensities, and numerous directions such as vertical, horizontal, or diagonal-for a 2D analysis and 13 directions for a 3D analysis are captured by GLCM. Entropy, which shows gray-level inhomogeneity or randomness, angular second moment, uniformity or energy, and contrast, draws attention to the gray-level differences between adjacent pixels or voxels that are some of the qualities that make up GLCM.


(2) $$\text{GLCM}(i,j,d,\theta \text{​})=\sum_{x=1}^{N}\sum_{y=1}^{M}\text{​}\{\begin{array}{ll} \text{​}1, & \text{​​​if }I(x,y)=i\text{ and }I(x+d,y+\theta \text{​})=j\\ 0, & \text{​​​otherwise} \end{array}$$

Galloway (1975) proposed the Gray Level Run Length Matrix (GLRLM), as shown in Equation (3), which is intended to record the spatial distribution of succeeding pixels in one or more directions, as well as in two or three dimensions. Many elements of GLRLM are included, such as fraction which examines the percentage of pixels in the Area of Interest. The availability of short and long runs is shown, respectively, by the weighted measurements known as short- and long-run emphasis inverse moments. Measures that evaluate the dispersion of runs over various gray levels and run lengths, respectively, are run-length non-uniformity and gray-level non-uniformity.


(3) $$\begin{array}{l} \text{GLRLM}(i,j,d,\theta )=\\ \sum_{x=1}^{N}\sum_{y=1}^{M}\{\begin{array}{ll} 1, & \text{if }I(x,y)=i\text{ and }I(x+d,y+\theta )=j\\ 0, & \text{otherwise} \end{array} \end{array}$$

An effective statistical method for characterizing textures is the gray-level size zone matrix (SZM), as shown in Equation (4), which was first presented by Thibault et al. (2013). The Gray Level Size Zone Matrix (GLSZM) uses the infinity norm to quantify the gray-level zones in an image. These are the areas where linked voxels have the same gray-level intensity within a given distance, usually 1. Texture homogeneity increases SZM's size and smoothness. While SZM does not require multidimensional calculations such as RLM and COM do, its efficacy is dependent on gray-level compression, therefore the best way to use it is to test out various compression techniques on training datasets.


(4) $$\begin{array}{l} \text{GLSZM}(i,j)=\\ \sum_{x=1}^{N}\overset{M}{\underset{y=1}{\sum }}\{\begin{array}{ll} 1, & \text{if }I(x,y)=i\text{ and the size of the zone containing }i\text{ is }j\\ 0, & \text{otherwise} \end{array} \end{array}$$

Based on their importance in collecting various facets of textural and structural information in medical tomography images, we chose these characteristics. Our objective was to achieve a balance between removing useful characteristics and avoiding too much dimensionality, which might result in overfitting and more complicated computations. The use of these elements enhances the overall efficacy of our suggested approach for image classification and enables us to provide a meaningful representation of the textures contained in the images.

### 5.2 Dataset

For our study, we utilized the MRI dataset provided by the Brain Tumor Segmentation (BraTS) Challenge in 2020 and 2021. The BraTS 2020 Challenge dataset is used to produce a separation model that could detect the malignancy region. The dataset comprised 369 MRI images captured in four distinct modalities, T2-weighted (T2w), T1-weighted (T1w), fluid-attenuated inversion recovery (FLAIR), and T1-weighted contrast-enhanced (T1wCE). These images and extraction patterns were provided in NIfTI format with coronal orientation. The masks provided four classifications, including non-tumor, non-enhancing tumor core, peritumoral edema, and enhancing tumor. As we were only interested in the broad tumor area, we combined the last three types for our analysis.

The Radiological Society of North America (RSNA) and the Medical Image Computing and Computer Assisted Intervention (MICCAI). Society expanded the MGMT promoter methylation detection component of BraTS challenge in 2021. A pre-selected collection of 585 MRI images from 2020 in almost the same four modalities was made available. To represent a broad spectrum of healthcare practices used across the world, the images were collected from several institutions utilizing a range of tools. For the classification task, we used these datasets, which were in DICOM format and annotated with their methylation status. The methylation pattern of MGMT was confirmed by laboratory analysis of surgical brain tumor tissues. The four modalities were kept the same as in the earlier datasets, but the T1w scans were not utilized since the diameters were continuously enormous, resulting in much more chaos than information.

### 5.3 Experimental setup

The proposed feature fusion model uses the Keras, PyTorch package with the TensorFlow backend and is entirely written in Python. Experiments are carried out utilizing MRI slices with a resolution of 256 × 256 to evaluate all of the suggested feature instrument networks. The cost function's relationship to its parameters must be optimized using stochastic scaling for the BraTS dataset of convolutional neural networks (CNNs). We utilized the adaptive moment (Adam) estimator for parameter estimation. Adam typically uses the first and second moments of the gradients to update and fix the linear trend derived from the real gradients. The settings for our Adam optimizer are as follows: 150 epochs is the maximum allowed, and the learning rate is 0.0001. With all biases set to 0, all weights are normally distributed with a mean of 0 and a variance of 0.01.

### 5.4 Dataset preprocessing

For each sample in the BraTS21 dataset, four NIfTI files containing different MRI modalities are provided. During the initial phase of data pre-processing, these modalities were stacked, resulting in an input tensor of shape (4, 240, 240, 155) in the (C, H, W, D) layout, where C represents the channels, H represents the height, W represents the width, and D represents the depth. Next, the redundant background voxels (with a voxel value of zero) at the edges of each volume were removed, as they did not provide any useful information and could be eliminated by the neural network.

The standard deviation for each channel was then calculated independently within the non-zero zone for each image. To normalize all volumes, the mean was first subtracted, and then, the standard deviation was divided. The background voxels were left unnormalized, and their measure remained at the cardinal. To differentiate between normalized voxels, which had numbers close to zero, and background voxels, an extra source channel was created using one-hot encoding for foreground voxels and then combined with the input data.

**Figure 4:**
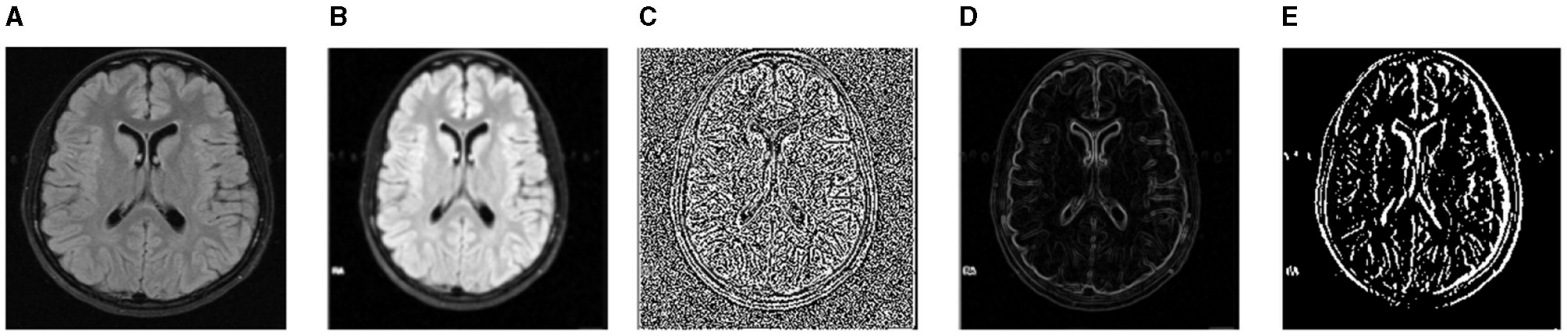
For noise removal, we apply different preprocessing techniques and acquire noise-free images of the BraTS dataset. **(A)** Input image. **(B)** SWMF Filter. **(C)** Laplacian. **(D)** Sobel. **(E)** Gabor.

Image segmentation is frequently completed as part of the image enhancement activity. The initial step in comprehending an image is to improve it. We used three datasets in the empirical results section: TCIA, FIGSHARE, and BraTS 2019. As a result, we will go over the pretreatment steps for each dataset in this section. We used initial image improvement approaches in the TCIA dataset: noise reduction and contrast adjustment. The artifacts caused by the imaging approach are attempted to be reduced using preliminary processing procedures. Additionally, noise reduction is a competent way to enhance outcomes before analysis (e.g., edge detection on image). It should be noted that the images used are gray-scale. We utilized two filters to remove noise: a median filter and a soft filter. After that, we will show you how to use the two filters. The median filter is a type of non-linear digital filter. It is a technique for removing unwanted signals or noise from an image. The Soft Weighted Median Filter (SWMF) is a novel image-processing approach for removing noise. Two noisy images are processed using this filter. The first is constant value noise, which is similar to salt and pepper noise in that its value does not vary. The second type is Random Value Noise (RVN), which is a sort of arbitrary value noise that has a variable value, similar to Gaussian and Speckle noise. The outcomes of the preprocessing steps are shown in Figure 4. The preprocessing techniques were only applied to the BraTS dataset.

The batch normalization approach is used on the FIGSHARE and BraTS datasets. Convolutional neural network (CNN) training is a complex task with several issues. Batch normalization is one of the most used methods for dealing with this problem. It is a widely used strategy in the field of deep learning. Batch normalization accelerates neural network learning. Moreover, batch normalization provides regularization, preventing over-fitting.

Data augmentation is a technique for avoiding over-fitting by artificially growing datasets during the learning stage. The essential data augmentations were used throughout the learning phase to strengthen our method:

1. Flips: the volume of each axis was reversed with a probability of 0.5 for the *x*, *y*, and *z* axes individually.
2. Gaussian Blur: The deviation of the Gaussian Kernel is obtained periodically from (0.5, 1.5) with a probability of 0.15, subjecting the reference volume to Gaussian fading.
3. Brightness: at a frequency of 0.15, a random number is regularly selected at random from the range (0.7, 1.3), after which source volume voxels are increased by it.
4. Zoom: the image size is increased to its initial dimensions twice the chosen value using cubic interpolation, and the source data are scaled using nearest neighbors interpolation. An arbitrary value is regularly gathered from (1.0, 1.4) with a frequency of 0.15.
5. Gaussian noise: every voxel is captured, and the source volume is then filled with randomized Gaussian noise with an average of zero and variance regularly selected from the range of (0, 0.33) with a probability of 0.15.
6. Contrast: at a frequency of 0.15, an arbitrary value is uniformly obtained from (0.65, 1.5). It is then enhanced, and the source volume voxels are trimmed to the value of the original range.
7. Biased crop: From the source volume, a piece with the dimensions (5, 128, 128, 128) was arbitrarily selected. Moreover, a probability of 0.4 ensures that some prominent voxels (with true positives in the underpinning data) will be retained in the trimmed area of the patch chosen using arbitrary-biased crop.

### 5.5 Multiple kernel for classification

For classification techniques, monitored learners such as the Support Vector Machine (SVM) method are utilized. We used an expanded form of SVM that includes various kernel training. The fundamental SVM operates as such. The content is first divided into binary categories. Next, a hyper-plane is located which distinguishes between both categories. Support vectors seem to be the parameters that seem to be close to the hyper-plane and are scientifically described in Equations (5–7):


(5) $$\begin{array}{l} \left(m,n\right),...\left(m_{i},n_{i}\right),...\left(m_{j},n_{j}\right);m_{i}\in R^{N},n_{j}\in \left\{-1,1\right\} \end{array}$$

The below expression can be used to represent the hyper-plane that categorizes a particular set of information as being linearly distinguishable. The Maximum Dividing hyper-plane is the name of this hyper-plane.


(6) $$\begin{array}{l} f\left(x\right)=\overset{j}{\underset{i=1}{\sum }}\left(\alpha_{i}n_{j}\left(X_{i}^{T}X\right)+b\right) \end{array}$$

Provided below is a depiction of the ideal hyperplane accompanied by the support vectors *m*_1_, *m*_2_, and *m*_3_ which are located on its edge.


(7) $$\begin{array}{l} g\left(\bar{X}\right)=\bar{W^{T}}\bar{X}+b \end{array}$$

A variety of techniques for traditional machine learning are used by numerous kernel systems. A predetermined collection of kernels is included in each approach. The kernel function allows the SVM to transform the data into a higher-dimensional space, facilitating the separation of data points through both linear and nonlinear decision boundaries. This strategy lessens the influence of bias throughout the training experience.

For the overall classification of BraTS, the precision and accuracy statistics of the best kernel operations are accumulated for all characteristics. Whereas the polynomial kernel is reliable for classifying radiomics-selected features, three different feature configurations (GLCM, GLRLM, and GLSZM) are better identified using the linear kernel function in SVM. Table 1 shows the top-chosen kernel technique for the BraTS sample together with assessment precision of the kernel function.

**Table 1:** We report the results of employing a Support Vector Machine (SVM) on the BraTS, TCIA, and FIGSHARE datasets to classify particular radiomics characteristics (GLCM, GLRLM, and GLSZM) in terms of sensitivity, specificity, precision, accuracy, and F1-score.

**Dataset**	**Feature set**	**Sensitivity**	**Specificity**	**Precision**	**Accuracy**	**F1-Score**
BraTS	GLCM	73.16	74.31	75.91	75.14	74.93
	GLRLM	71.36	71.85	72.34	72.76	73.44
	GLSZM	73.69	71.94	73.49	72.35	73.11
	Average	72.74	72.7	73.91	73.43	73.83
TCIA	GLCM	75.20	73.80	74.50	74.75	74.25
	GLRLM	74.40	72.90	73.80	73.15	73.60
	GLSZM	73.60	75.10	74.90	74.35	74.75
	Average	74.40	73.60	74.07	74.08	74.20
FIGSHARE	GLCM	74.71	76.05	77.13	76.58	76.81
	GLRLM	72.85	73.42	74.12	73.64	73.98
	GLSZM	76.07	74.91	75.94	75.49	75.72
	Average	74.04	74.46	75.06	75.24	75.50

Included are the average performance metrics for every dataset.

By using the SVM classifier on the BraTS, TCIA, and FIGSHARE datasets, we subsequently trained and validated UNet, VNet, and UNet^++^ on a classification task that incorporated.

In five-fold cross-validation, the classification algorithm we created using a Support Vector Machine with different Kernels yielded an estimated ROC AUC of 96.4 ± 0.43%. On a fusion features classification assignment, we trained different pre-trained CNN models such as AlexNet, SqueezeNet, VGG16, InceptionV3, Xception, UNet, VNet, and UNet^++^, as well as a workflow using the categorization predictions of these models against a professional investigator. The pipeline increased recall (93.4 ± 0.5% vs. 95.4 ± 0.7%) without noticeably reducing accuracy (81.5 ± 0.4% to 95.7 ± 0.5%).

Our findings show that UNet and other similar networks such as VNet and UNet^++^ continue to have large false positive rates, which may preclude their application in healthcare situations. We showed that adding a different classifier significantly increases accuracy. We are aware that this is simply one of many viable options; future advancements to UNet could eliminate the requirement for a different classifier.

## 6 Result and discussion

This is the first study that, as far as we know, combines radiomic analysis for medical imaging with deep neural network installation. Our findings show that utilizing transpose convolution for both up and downsampling, the integration of Seq-ResNet architecture greatly enhances the ability to identify cancerous slices in brain MRI images. This new mix of customized imaging biomarkers and powerful deep learning approaches boosts model performance on the BraTS dataset, even with a smaller patient cohort compared with earlier research. We carried out effective image preprocessing to guarantee reliable and repeatable deep learning execution. To be more precise, we scaled each image to a 256 × 256 grid and normalized it to 256 gray levels. The repeatability of our findings is aided by these common digital image processing procedures. Furthermore, we carried out a thorough assessment of the pre-trained CNN models' dependability. The promise for enhanced medical image analysis is highlighted by this fusion of complex neural network topologies with exact image preprocessing techniques, opening the door to more accurate brain tumor diagnosis.

Average Precision (AP), mean Average Precision (mAP), and F1 are utilized in the trials to assess the effectiveness of the suggested framework. Finding objects and classifying them into multiple classes is the basic goal of image analysis. The assessment metrics for these two tasks are recall (abbreviated as R) which may be stated as “the percentage of the appropriate tumors identified to all tumors” and precision, “the accurate rate of the categorization of identified tumors” (abbreviated as P). The terms False Positive (FP), False Negative (FN), True Positive (TP), and True Negative (TN) are used to characterize these metrics. The calculation for Precision (P) and Recall (R) is shown in Equation (8):


(8) $$\begin{array}{c} \begin{array}{cc} R & =\frac{TP}{TP+FN}\\ P & =\frac{TP}{TP+FP} \end{array} \end{array}$$

The accuracy and recall metrics might tend to be in conflict when measuring the results of several architectures. Additionally, a single index must be used to determine accuracy of a classifier. Precision and Recall create a rectangle-coordinated graph using Precision and Recall as the coordinates after being ordered by grading value. Precision-Recall curve is the name of this rectangle-shaped graph. The area underneath the Precision-Recall curve or AP is the average of APs across many classes or mean Average Precision (mAP). The Equation (9) mAP evaluates the classifier's performance across all classes, AP evaluates the classifier's performance across each category. As a result, in this study, AP is employed when goals come from a single class, whereas mAP is utilized when criteria come from a variety of categories. Another often-used indication for object detectors is F1-Measure (sometimes called F1-Score). Recall (R) and Precision (P) are weighted averaged to get the F1-Measure. F1-Measure is referred to as F1 when it is equal to 1:


(9) $$\begin{array}{c} \begin{array}{cc} F1 & =\frac{\left(\alpha^{2}+1\right)\times \left(P\times R\right)}{\alpha^{2}\left(P+R\right)}\\ F1 & =\frac{2\times \left(P\times R\right)}{P+R}\\ mAP & =\frac{1}{N}\overset{N}{\underset{i=1}{\sum }}AP_{i} \end{array} \end{array}$$

A higher F1 score denotes greater classifier efficiency. The F1 score is calculated by combining the Precision and Recall values. We also offer the Recall and Precision values of the suggested approach on several datasets to give a thorough evaluation. Within a transfer learning framework, this study uses eight popular Convolutional Neural Network (CNN) pre-trained models. Since VGG16 has a relatively modest number of learnable parameters under the transfer learning framework and is widely used in medical image evaluation tasks, it was first selected because it required less computing power for network training than other well-known models.

For the proposed radiomic-based FusionNet architecture, we also looked at AlexNet, SqueezeNet, VGG16, InceptionV3, Xception, UNet, VNet, and UNet^++^. In contrast, the efficiency after the feature fusion architecture was greater in the UNet pre-trained model than in the VNet model. This difference can be attributed to the superior classification results of UNet utilizing solely MRI slices compared with VNet. The empirical analyses (precision, specificity, recall, sensitivity, F1, and accuracy) of experimental pre-trained CNN models with radiomics features are shown in Table 2. The radiomics feature fusion with deep learning model produced substantial gains in all parameters with *p* ≤ 0.05 for the pre-trained CNN architecture and proposed FusionNet model. Furthermore, the data with lower standard deviations demonstrated the radiomics feature fusion with deep learning architecture's improved resilience. These quantifiable findings demonstrate the efficiency of the proposed feature fusion with the deep learning model.

**Table 2:** Training accuracy, F1-Score, specificity, precision, and sensitivity on the BraTS, TCIA, and FIGSHARE datasets of the proposed model (FusionNet with ResNet, FusionNet with Seq-ResNet, and FusionNet with Seq-ResNet and radiomics features) and other pre-trained CNN models such as AlexNet, SqueezeNet, VGG16, InceptionV3, Xception, UNet, VNet, and UNet^++^.

**Dataset**	**Model**	**Sensitivity**	**Specificity**	**Precision**	**Accuracy**	**F1-Score**
BraTS	AlexNet	81.39	81.43	82.04	81.93	81.73
	SqueezNet	82.19	83.29	82.95	83.07	82.78
	VGG16	82.63	82.14	83.09	82.79	82.54
	InceptionV3	84.95	85.23	85.31	85.49	85.19
	Xception	85.36	85.94	85.97	85.43	85.27
	UNet	86.49	87.09	87.12	86.71	86.34
	VNet	86.91	86.97	87.04	87.12	87.01
	UNet^++^	88.13	88.27	87.38	87.51	87.21
	FusionNet	**90.19**	**89.77**	**89.84**	**90.07**	**89.92**
	FusionNet-Seq-ResNet	**93.06**	**93.34**	**93.41**	**93.79**	**93.81**
	FusionNet-Seq-ResNet+Radiomics	**95.19**	**95.37**	**95.46**	**95.83**	**95.79**
TCIA	UNet	82.34	82.78	83.02	82.69	82.88
	VNet	83.12	83.45	83.21	83.34	83.17
	UNet^++^	81.87	81.98	82.45	82.13	82.28
	FusionNet	**91.56**	**91.23**	**91.92**	**91.64**	**91.74**
	FusionNet-Seq-ResNet	**93.02**	**93.71**	**93.19**	**93.93**	**94.09**
	FusionNet-Seq-ResNet + Radiomics	**95.07**	**95.84**	**94.98**	**94.93**	**95.16**
FIGSHARE	UNet	86.21	86.45	86.32	86.39	86.28
	VNet	87.03	86.89	87.12	87.08	87.17
	UNet^++^	87.45	87.51	87.38	87.62	87.49
	FusionNet	**91.67**	**92.78**	**92.45**	**91.72**	**92.59**
	FusionNet-Seq-ResNet	**94.21**	**94.34**	**94.47**	**93.15**	**93.28**
	FusionNet-Seq-ResNet+Radiomics	**95.17**	**95.81**	**95.67**	**95.72**	**95.86**

A significant degree of resilience is shown by the variance, which is < 3% after 50 epochs. When comparing the pre-trained CNN models with the pilot model, the average values of all parameters for the suggested FusionNet architecture are superior. Only a few statistically significant improvements are observed in the MRI tumor and healthy class data, and the reported mathematical benefits are minor. Further evidence of enhanced resilience is provided by the FusionNet model's decreased variances.

**Figure 5:**
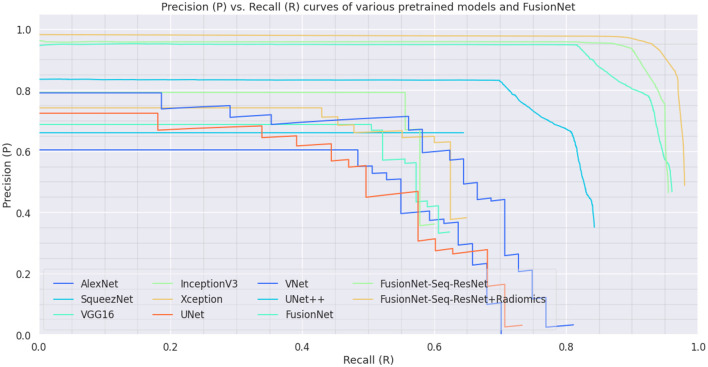
Precision-Recall Curve on BraTS dataset: Precision-Recall curve of the proposed model (FusionNet with ResNet, FusionNet with Seq-ResNet, and FusionNet with Seq-ResNet and Radiomics features) and other pre-trained CNN models such as AlexNet, SqueezeNet, VGG16, InceptionV3, Xception, UNet, VNet, and UNet^++^.

On the BraTS dataset, the performance and efficacy of the novel suggested framework have been tested and verified. Table 2 displays the Average Precision of the proposed Fusion CNN model framework together with other cutting-edge and pre-trained CNN models. The novel and proposed framework, as shown in Figure 2, obtains an AP of 97.53%, which is ~3.7% higher than the APs attained by other pretraind CNN models such as AlexNet, SqueezeNet, VGG16, InceptionV3, Xception, UNet, VNet, and UNet^++^. The proposed FusionNet classifier's Recall and Precision curve and specific performance are shown in Table 2 and Figure 5.

To exhibit the performance and efficacy of the novel proposed Seq-ResNet with FusionNet, upsampling and downsampling have been evaluated and the results are shown in Figure 5 and Tables 2, 3.

**Table 3:** Training accuracy, F1-Score, specificity, precision, and sensitivity on the BraTS dataset of the proposed model (FusionNet with ResNet, FusionNet with Seq-ResNet, and FusionNet with Seq-ResNet and radiomics features) and other state-of-the-arts methodologies.

**Model**	**Sensitivity**	**Specificity**	**Precision**	**Accuracy**	**F1-Score**
Zare et al. (2018)	81.39 ±	–	–	82.11 ±	–
Zhu et al. (2023)	92.00 ±	93.11 ±	92.01 ±	92.03 ±	–
Jie et al. (2023)	91.17 ±	–	–	92.04 ±	–
Wen et al. (2023)	86.15 ±	–	–	87.12 ±	86.77 ±
Singh and Anand (2019)	–	87.15	–	87.11	88.15
Qin et al. (2018)	–	–	–	77.80	–
Dogra and Kumar (2022)	87.16	–	–	87.08	88.15
Wang et al. (2021)	89.13	88.71	87.19	87.11	88.15
FusionNet	**90.19**	**89.77**	**89.84**	**90.07**	**89.92**
FusionNet-Seq-ResNet	**93.06**	**93.34**	**93.41**	**93.79**	**93.81**
FusionNet-Seq-ResNet+Radiomics	**95.19**	**95.37**	**95.46**	**95.83**	**95.79**

FusionNet with radiomics features uses Sequential Residual Network layers to create its backbone in contrast to the planned FusionNet (which uses conventional ResNet layers), although other components are the same. In other respects, the Seq-ResNet employed in their backbones is the only distinction between simple FusionNet and FusionNet with Seq-ResNet. By contrasting the assessment outcomes of FusionNet and FusionNet with Seq-ResNet, it is possible to show the usefulness of the suggested novel Seq-ResNet layers. FusionNet without Seq-ResNet obtains an AP of 94.73%, which is 3.31% lower than the AP achieved by FusionNet with Seq-ResNet, according to the assessment findings shown in Tables 2, 3. To put it another way, Sequential Residual Network (Seq-ResNet) layers outperform simple ResNet in the evaluation by 3.31% AP.

Another major difference between simple FusionNet and FusionNet with Seq-ResNet is using upsampling and downsampling modules. By contrasting the assessment outcomes of the proposed simple FusionNet and FusionNet with Seq-ResNet, it is possible to show the usefulness of the proposed FusionNet with Seq-ResNet. Table 2 shows that the feature fusion with upsampling and downsampling modules delivers a strong performance advantage of 3.18% AP when compared with normal simple FusionNet. To have a thorough grasp of the proposed novel FusionNet with Seq-ResNet performance, the classification results are carefully examined. The majority of tumors may be appropriately located in the classification outcomes irrespective of their orientations, colors, and scales, proving the usefulness of the suggested framework. However, the suggested technique occasionally fails when portions of the tumors make up more than half of the original kernel values.

The BraTS dataset has also assessed the proposed framework. Table 2 displays the assessment outcomes of the proposed FusionNet with Seq-RestNet and other pre-trained CNN models such as AlexNet, SqueezeNet, VGG16, InceptionV3, Xception, UNet, VNet, and UNet^++^. Generally speaking, the suggested novel framework outperforms existing state-of-the-art algorithms, achieving an F1 Score of 96.72%.

Figure 5 shows the Precision and Recall curves for the proposed FusionNet with Seq-ResNet framework based on the BraTS dataset. The comprehensive assessment findings for the suggested novel framework are shown in Table 2. Table 4 shows that the proposed FusionNet with Seq-ResNet performs well for identifying tumors in MRI and achieves plausible results when detecting tumors in MRI images with F1 Score of 95.88 and 96.72%, respectively. As compared with other algorithms, the performance of FusionNet with Seq-ResNet can be steady even as the tumor's scales change quickly.

**Table 4:** FusionNet training accuracy, F1-Score, specificity, precision, and sensitivity on the BraTS dataset.

**Fold**	**Sensitivity**	**Specificity**	**Precision**	**Accuracy**	**F1-Score**
Fold-1	93.29	94.71	95.81	95.57	94.38
Fold-2	94.37	95.13	95.77	96.51	95.04
Fold-3	95.91	95.31	96.85	96.17	95.64
Fold-4	94.88	95.57	96.15	97.13	95.89
Fold-5	95.89	95.21	97.77	97.53	96.12
Average	94.88	95.18	96.47	96.58	95.41

The accuracy, precision-recall, and F1-measure calculations are used to examine the quantifiable efficiency. The performance and efficiency measures for tumor detection (Accuracy, Sensitivity, Specificity, Precision, and F1 measure) for the dataset BraTS are shown in Table 2. The table shows that the suggested technique performs better than previous methods in terms of Precision-Recall and F1 measure. The suggested technique combines many aspects that are better able to depict image changes and hence execute both statistically and aesthetically better. It is frequently used as a plot indicative of the effectiveness of the classifier. A ROC curve is a useful tool for visualizing, organizing, and choosing learners based on their efficiency. A classifier's ability to determine outcomes is measured by the Receiver Operating Characteristic (ROC), which contrasts and illustrates the trade-off between the model's specificity and sensitivity. The result for the ROC plot is the region beneath the ROC curve, and a large value denotes a successful algorithm. An excellent predictor produces a value in the top left corner of the ROC space, or coordinate (0,1), signifying 100% specificity (zero false positives) and 100% sensitivity (zero false negatives). The ROC plot for the BraTS dataset for the various change detection methods is shown in Figure 7. It is evident from the chart that for the dataset, the suggested strategy outperforms conventional approaches. The area under the ROC curve or AUC is used to calculate the ROC plot's mathematical value. A high AUC value means the applied tumor detection framework is good and can successfully differentiate between benign and malignant patches. By examining the proximity of the curve to the top left corner of the image in Figure 7, it is evident that the suggested strategy works better than the established tumor detection methods for the BraTS dataset.

**Figure 6:**
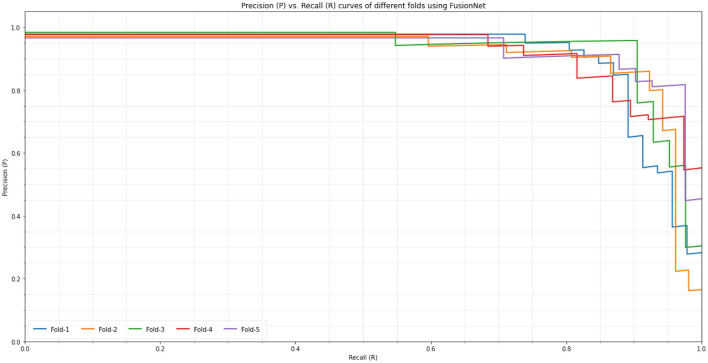
Precision (P) vs. Recall (R) curves of different folds using proposed model (FusionNet) on the BraTS dataset.

In Figure 5, the novel proposed method's precision-recall outcomes are shown. For MRI images, the first fusion experiment was conducted. The radiomics feature fusion with deep learning features approach offers more accurate anatomical details in MRI images separately. Figure 5 shows how the suggested strategy maintains the measurement elements of MRI images in the fused images. The study that performed on the proposed FusionNet model in consideration of various metrics under various numbers of folds is shown in Table 4 and Figure 6. The proposed FusionNet model's specificity and sensitivity assessments for various fold numbers are shown in Figure 6. The greatest specificity and sensitivity outcomes for the novel proposed FusionNet model under fold-1 is 94.71 and 93.29%, respectively. The presented feature fusion model also produced higher specificity and sensitivity results under fold-2, with corresponding outcomes of 95.13 and 94.37%, respectively. The presented feature fusion model approach also produced the highest specificity and sensitivity outcomes under fold-3, which were 95.31 and 95.91%, respectively. Additionally, the reported FusionNet model strategy had higher specificity and sensitivity under fold-4 95.57 and 94.88%, respectively. The suggested feature fusion approach also showed ideal specificity and sensitivity results of 95.89 and 95.21%, respectively, under fold-5.

**Figure 7:**
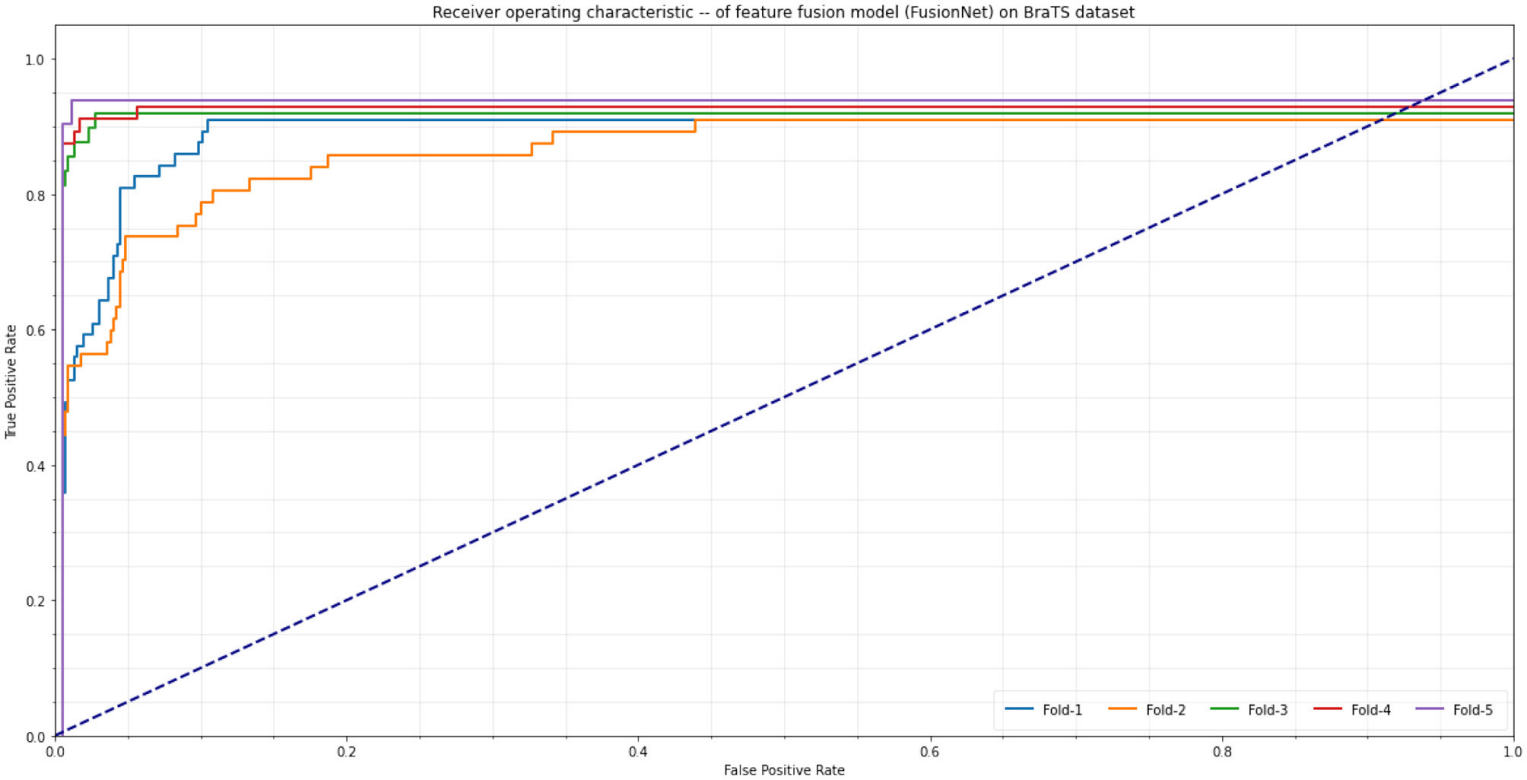
Receiver Operating Characteristic (ROC) curves of different folds using proposed model (FusionNet) on the BraTS dataset.

The ROC curves of the proposed FusionNet model are shown in Figure 7, demonstrating its accuracy and precision at different fold counts during cross-validation. The model performs better than expected, with fold-1 obtaining 95.57% accuracy and 95.81% precision by combining feature fusion with up and downsampling methods. The feature fusion method yields the best accuracy and precision in fold-2, with 96.51 and 95.77%, respectively. Transpose Convolution, when used for up and downsampling in fold-3, produces even better results, with accuracy and precision reaching 96.17 and 96.85%, respectively. The model achieves significantly greater accuracy and precision under fold-4, 97.13 and 96.15%, respectively. The feature fusion model under fold-5 performs best overall, with a precision of 97.77% and an accuracy of 97.53%. This shows how well the suggested FusionNet integrates deep learning methods for improved medical image interpretation.

A novel framework for the CAD solution may be established by the proposed architecture FusionNet comprises Seq-ResNet and up-down sampling with Transpose Convolution, which combines radiomics analysis with Convolutional Neural Network implementation. The established Seq-ResNet calculation method may potentially improve the efficiency of neural networks in other applications, especially those requiring the intake of multi-channel imaging images. The suggested technique also offers a radiomics viewpoint on the interpretability of deep learning. Since the neural network's hyperparameters were developed without explicit personal expertise interaction, actual interpretation of them is challenging. The black box world of deep learning-based CAD systems hindered their medical and clinical implementations without the support of medical practitioners and radiologists. We explored neural network information using a radiomics-based approach as a first milestone toward deep learning interpretability. It has been shown that radiomic feature regions may be computationally fragmented to produce interpretation. Radiomics have been extensively researched as computational imaging biomarkers for illness identification and performance assessment. Complicating factors, such as anatomical outlines from radiation therapy and histopathology samples from biopsy, can be employed to improve deep learning interpretability after the saliency analytical method in this study. Future research will examine these topics once suitable datasets are obtained.

## 7 Ablation studies and future works

We performed several sets of ablation experiments to demonstrate the effectiveness of the suggested vascular segmentation method. The purpose of these tests was to investigate the effects of different loss parameter settings and the efficacy of different techniques. We create a baseline using an X-shaped network that contains an encoder with modified sequential residual blocks and a feature decoder (influenced by Szegedy et al., 2017), to validate the efficiency of the proposed methodologies. We develop our network based on this foundation. Tables 2, 3 and Figure 5 show the results, from which we derive several inferences.

Table 2 shows the quantitative experiment data for the baseline and the three proposed models. When compared with the baseline, Model 1 (FusionNet) improves Accuracy, F1-Score, Sensitivity, Specificity, and Precision by 2.15%, 1.89%, 1.96%, 1.73%, and 2.11% percent, demonstrating the benefits of using both up and downsampling strategies. Furthermore, when compared with FusionNet, FusionNet-Seq-ResNet achieves a 2.94% improvement in Accuracy, while FusionNet-Seq-ResNet + Radiomics achieves a 4.81% improvement, demonstrating the efficacy of the Sequential Residual Network and radiomics features. FusionNet exceeds UNet by 2.79% in F1-Score, demonstrating that combining up and downsampling approaches improves the model's performance even further. Similarly, when compared with UNet++, FusionNet-Seq-ResNet shows a 2.98% gain in F1-Score, highlighting the importance of Transpose Convolution and deconvolution in improving brain MRI classification. Finally, when compared with VNet, FusionNet-Seq-ResNet + Radiomics improves accuracy by 3.97%, highlighting the effectiveness of radiomics features and the impact of Transpose Convolution and deConvolution in up and downsampling. The proposed approaches exhibit significant improvements over the baseline, with increments of 3.98% in accuracy, 4.15% in F1-score, 3.89% in Sensitivity, 3.91% in Specificity, and 3.79% in Precision, demonstrating the proposed network's superior segmentation performance.

Our new local convolution-based network, FusionNet, performs well on publicly accessible BraTS datasets. We do, however, find a constraint on its ability to generalize to other data distributions. This restriction results from the fundamental characteristics of local convolution, which prioritize local data above important global data. Consequently, long-term dependencies are difficult for CNNs to capture and are crucial for improving the model's summarization ability. We investigate the possibility of using the Transformer design, which has demonstrated efficacy in creating global dependencies via a self-attention mechanism, to tackle this problem. Transformers have several drawbacks, including a high parameter count and a heavy dependency on large amounts of training data, even if they provide dynamic attention and global context fusion.

We track the variations in accuracy as the epochs proceed during the training process of various networks. The results are shown in Figures 6, 7. When comparing FusionNet with standard pre-trained CNN networks and a network created specifically for BraTS classification, it is clear that FusionNet has easier convergence and training. This gain can be ascribed to FusionNet's Seq-ResNet module, which improves learning ability while utilizing much fewer parameters than typical convolutional layers. When trained on 1,315 samples, the ROC of FusionNet reaches 0.79 at the 13th epoch, 0.86 at the 27th epoch, and 0.885 at the 35th epoch, as shown in Figure 7. On an NVIDIA 1050TI, training for one epoch takes 297 s. This suggests that by applying FusionNet, a high-performing BraTS MRI classification model may be produced in 137 min (80 epochs). These findings confirm that the proposed FusionNet is simple to train and capable of giving satisfactory outcomes.

Table 2 shows how incorporating the up and downsampling module into FusionNet improves performance. FusionNet beats pre-trained CNN models, improving accuracy from 87.11 to 90.07%, precision from 88.15 to 89.92%, and F1-Score from 87.38 to 89.84%. These improvements are ascribed to the proposed up and downsampling module's dynamic merging of multi-scale context information. Two experiments were carried out to further validate this. First, we introduced a Seq-ResNet module, FusionNet-Seq-ResNet, into the baseline with no point-wise convolution rate. It improved accuracy by 4.21% over the baseline but decreased by 2.18% when compared with FusionNet-Seq-ResNet + Radiomics. This result emphasizes the need to obtain multi-scale contextual information. Second, we incorporated radiomics features with various Transposed convolution and deconvolution settings into FusionNet's parallel branches. While these two models outperformed the baseline and FusionNet-Seq-ResNet, they fell just short of FusionNet. This finding demonstrates that the FusionNet-Seq-ResNet + Radiomics transposed convolution and deConvolution configuration is best, and the addition of multi-scale context information is especially useful for up and downsampling. As shown in Figure 6, FusionNet-Seq-ResNet +radiomics outperforms FusionNet in the analysis of under-segmented patches, particularly those with relatively small scales. This finding adds to the evidence that dynamic selection of multi-scale contextual information promotes more successful MRI analysis.

In the future, we suggest a hybrid network design that combines the best features of Transformers and CNNs. This method entails sandwiching a thin transformer module between the encoder and decoder of the CNN. By combining multi-scale information, the lightweight transformer will facilitate the effective integration of multi-scale information and global channel attention, spatial attention, and scale attention. The objective is to create a network that combines the benefits of Transformers (dynamic attention and improved generalization) with CNNs (local receptive fields, shared weights, shift, and scale invariance). By combining these elements, we hope to create a segmentation technique that preserves the advantages of both designs, enhancing FusionNet's overall robustness and speed.

## 8 Conclusion

According to the results of this comprehensive research, radiomics feature fusion with deep learning features in medical image analysis is a nascent but promising topic that supports medical practice in medical imaging interpretation across all disciplines. We have honed in on important insights, described unanswered problems and summarized essential terminology, approaches, and appraised the state of the art for radiomics feature fusion with deep learning features in medical imaging. Different preprocessing methodologies are carried out initially to continue improving the accuracy of the diseased patch in the proposed fusion framework (FusionNet), and the training dataset is subsequently employed to expand the training dataset. Various pre-trained learning models are used to design and train on the BraTS dataset. Additionally, a FusioneNet deep model is improved with radiomic features, and this manual feature is carried out with other models. Subsequently, the proposed fusion technique (FusionNet) is employed to better integrate the information rather than the initial serial-based methodology. A novel feature simplification approach is presented as a result of the examination of the fused feature space, which shows several duplicate characteristics. The proposed FusionNet model captures the structural, textural, and statistical aspects of brain tumors with an F1 score of 96.72, sensitivity and specificity of 96.31, and AUC of 96.93.

The topic of feature fusion for deep learning in medical imaging is growing, and it is anticipated that new fusion techniques will be created. The upcoming study should concentrate on common nomenclature and measurements for particular and appropriate direct evaluation of various radiomics fusion models. We discovered that radiomics feature fusion with deep learning features for automated medical imaging tasks significantly outperforms in single modality models, and further research may provide insights to guide the most effective methods. We will monitor the effectiveness of the presented methodology based on distinct handcrafted feature fusion by including spatial information and other medical datasets using deep learning and Seq-ResNet in the future.

## Data availability statement

Publicly available datasets were analyzed in this study. This data can be found at: https://www.med.upenn.edu/cbica/brats2020/data.html.

## Author contributions

SI: Writing – review & editing, Writing – original draft, Visualization, Methodology, Conceptualization. AQ: Writing – review & editing, Supervision, Software, Investigation, Formal analysis. MAl: Writing – review & editing, Visualization, Resources, Project administration, Funding acquisition. KA: Writing – original draft, Methodology, Formal analysis, Data curation. IC: Writing – original draft, Investigation, Formal analysis, Data curation, Conceptualization. MAn: Writing – original draft, Resources, Methodology, Formal analysis, Data curation.
